# Following the guidelines for communicating commensurate magnetic structures: real case examples

**DOI:** 10.1107/S2052520624005407

**Published:** 2024-07-17

**Authors:** F. Damay

**Affiliations:** a Université Paris-Saclay, Laboratoire Léon Brillouin, CEA-CNRS UMR 12 Gif-sur-Yvette 91191 France; Universidad del País Vasco, Spain

**Keywords:** commensurate magnetic order, magnetic space groups, magCIF standards, magnetic ordering, irreducible representations

## Abstract

A few real case examples are presented on how to report magnetic structures, with precise step-by-step explanations, following the guidelines of the IUCr Commission on Magnetic Structures [Perez-Mato *et al.* (2024). *Acta Cryst*. B**80**, 219–234]. Four examples have been chosen, illustrating different types of single-**k** magnetic orders, from the basic case to more complex ones, including odd-harmonics, and one multi-**k** order. In addition to acquainting researchers with the process of communicating commensurate magnetic structures, these examples also aim to clarify important concepts, which are used throughout the guidelines, such as the transformation to a standard setting of a magnetic space group.

## Abstract 2

Different examples are described on how to follow the recently published guidelines [Perez-Mato *et al.* (2024). *Acta Cryst*. B**80**, 219–234] when reporting a commensurate magnetic structure.

## 1. Introduction

With the increasing number of magnetic structure reports in the literature, standardization in the description of a magnetic structure has become a real need. Such standardization is the aim of the new report of the IUCr Commission on Magnetic Structures (CMS) ‘Guidelines for communicating commensurate magnetic structures’ (Perez-Mato *et al.*, 2024 ▸), published in this special issue. These guidelines rely on the existing magCIF format which, similar to the CIF format, contains all necessary information on the magnetic symmetry group, written in a standard form. This information can be easily retrieved to report a magnetic structure using its magnetic space group. The magCIF format is nowadays implemented in most of the computer resources available for the determination of a magnetic structure (Stokes *et al.*, 2020 ▸; Perez-Mato *et al.*, 2015 ▸; Rodríguez-Carvajal, 1993 ▸; Petříček *et al.*, 2023 ▸; Toby & Von Dreele, 2013 ▸), making it an accessible and useful tool to anyone working with magnetic structures.

To familiarize users with the process of reporting a commensurate magnetic structure in a standard way, this article explicitly details, for several real case examples, the information that is needed. Four examples have been chosen. The first example compares two types of **k** = (0, 0, 0) magnetic orderings in rare-earth pyrochlores: the straightforward case of a single-**k** order corresponding to a one-dimensional irreducible representation in Nd_2_Zr_2_O_7_, and a single-**k** order corresponding to a three-dimensional irreducible representation in Tb_2_Sn_2_O_7_. The primary aim of these two examples is to acquaint the user with magCIF information, in particular with respect to the transformation to a standard setting when using a magnetic space group. Along those lines, the second example illustrates how a magnetic space group label is meaningless without its transformation to standard setting, in the single-**k** [**k** = (½, 0, 0)] magnetic ordering of BiMnTeO_6_, where the magnetic space group *P_a_*2_1_/*c* can describe four different magnetic orders if no standard setting transformation is provided. Different magnetic space group notations are also described in this example, including the new UNI standard. In the third example, an illustration is given of how the magnetic space group description accounts for harmonics in the magnetic ordering of BaMnO_3_ [**k** = (⅓, ⅓, 0)], and how this can be reported. The last example describes the multi-**k** case of TbCrO_3_, to which magnetic symmetry can be applied as easily as for a single-**k** order; this example also shows how to combine the magnetic space group and basis vectors to determine a magnetic structure efficiently, when more than one irreducible representation is involved in the magnetic transition.

These examples should facilitate the reporting of new magnetic structures following the criteria of the guidelines, and should also motivate authors to provide magCIF files as supporting information. The importance of uploading any new published magnetic structure in MAGNDATA (Gallego *et al.*, 2016 ▸) should also be underlined here, as any new entry will strengthen this database as a tool for the solid state science community.

## 2. Experimental

Magnetic symmetry analysis for the examples given in this article was performed using the software tools available on the Bilbao Crystallographic Server (BCS; https://www.cryst.ehu.es/), especially the *k-Subgroupsmag* and *Get_mirreps* routines (Perez-Mato *et al.*, 2015 ▸, 2016 ▸), in addition to *MPOINT*, *MTENSOR* and *MAGNDATA* (Gallego *et al.*, 2016 ▸). The group–subgroup graphs, and the directions of the order parameters of irreducible representations when mentioned, were also generated on the BCS.

Irreducible representations (IRs) with their basis vectors (also called spin basis modes) were obtained either from the *ISOTROPY* suite (*ISODISTORT*) (Stokes *et al.*, 2024 ▸, https://stokes.byu.edu/iso/isotropy.php; Campbell *et al.*, 2006 ▸) or from the *BasIrreps* routine of *FullProf* Suite (Rodríguez-Carvajal, 1993 ▸).

The notation for IRs follows the Cracknell–Davies–Miller–Love (CDML) notation (Cracknell *et al.*, 1979 ▸), with the letter ‘m’ indicating the odd character with respect to time reversal of the representation (magnetic representation).

Magnetic space group (MSG) notation follows the Belov–Neronova–Smirnova (BNS) standard, which, in several of the examples, coincides with the new unified (UNI) standard (Campbell *et al.*, 2022 ▸), as they are type-III MSGs [for the definition of the four different types of magnetic space groups, one can refer to Litvin (2001 ▸), and more recently to Campbell *et al.* (2022 ▸), for instance]. For the MSG of type-IV BiMnTeO_6_, BNS and UNI notations are given. The term grey (or paramagnetic) group used in the article refers to type-II MSGs, which describe MSGs containing time reversal as an operator. Following UNI notation, the time-reversal operation 1’ (or 1′) is separated from the rest of an MSG symbol by a period (.) for readability purposes. This notation is applied throughout the article whenever applicable, except on the BCS generated group–subgroup graphs.

All magnetic structures were drawn using *Mag2Pol* (Qureshi, 2019 ▸), which allows the import of the .mcif file generated by *k-Subgroupsmag*.

In all the examples presented here, the description of the (non-magnetic) crystal structure comes from the same reference as that cited for the description of the magnetic order.

All examples are also listed in the BCS MAGNDATA database of magnetic structures: Nd_2_Zr_2_O_7_ (entry No. 0.340), Tb_2_Sn_2_O_7_ (entry No. 0.48), BiMnTeO_6_ (entry No. 1.301), BaMnO_3_ (entry No. 1.0.39), TbCrO_3_ (entry Nos. 0.354 and 2.62). Corresponding magCIF files can be downloaded from this database for crosschecking or training purposes.

### 2.1. Examples

#### 2.1.1. Single k = (0, 0, 0) magnetic orders in rare-earth pyrochlores

*A*_2_*B*_2_O_7_ pyrochlores (*A* = rare-earth, *B* = tetravalent element) crystallize as a cubic 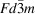 (No. 227) structure, with the magnetic rare-earth *A*^3+^ on Wyckoff position 16*d* (½, ½, ½). The *B*^4+^ atom is on 16*c* (0, 0, 0) and there are two independent oxygen atoms, O1 on the 8*b* position (⅓, ⅓, ⅓) and O2 on the 48*f* position 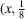, 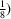.

**Figure 1:**
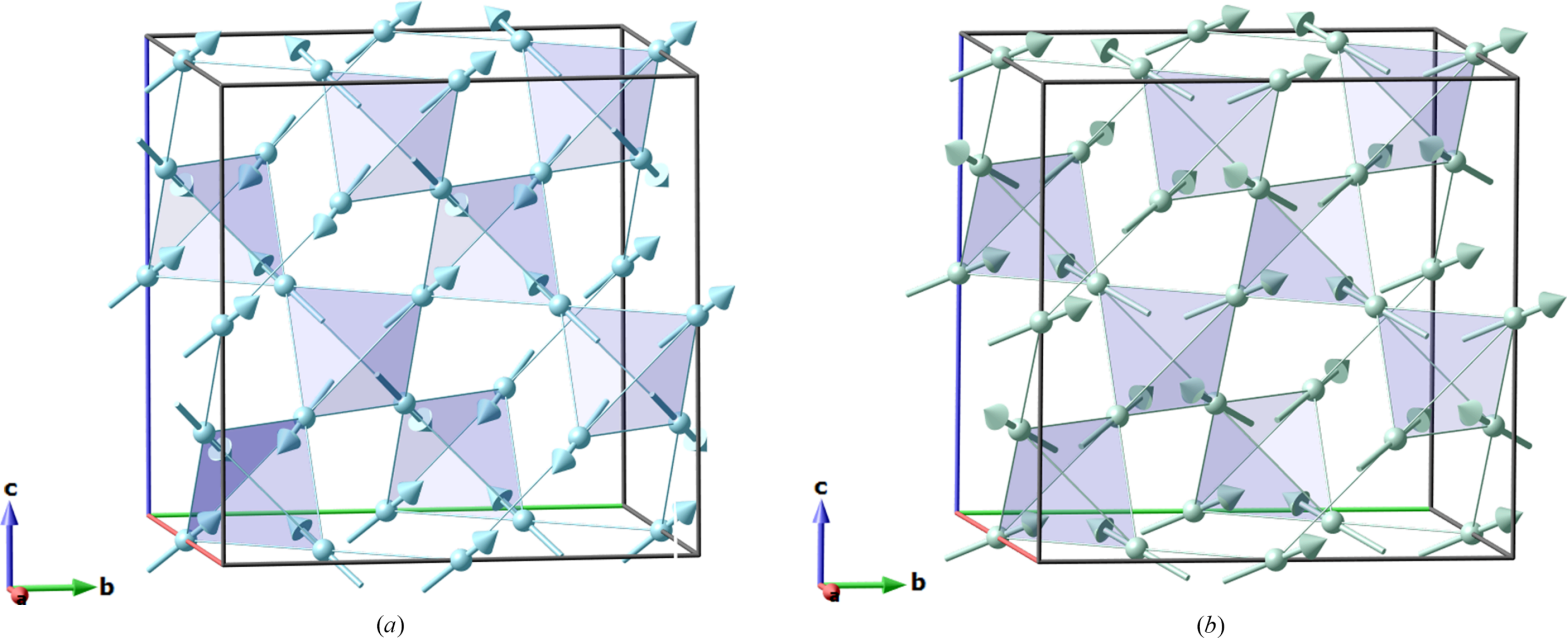
(*a*) All-in all-out magnetic order in pyrochlore Nd_2_Zr_2_O_7_ (Xu *et al.*, 2015 ▸; Lhotel *et al.*, 2015 ▸). (*b*) Two-in two-out magnetic order in Tb_2_Sn_2_O_7_. Only magnetic atoms (*A* site of the pyrochlore crystal structure) and their network of corner-sharing tetrahedra are shown.

In this structural family, Nd_2_Zr_2_O_7_ orders below the Néel temperature *T*_N_ = 300 mK (Lhotel *et al.*, 2015 ▸; Xu *et al.*, 2015 ▸) with a **k** = (0, 0, 0) magnetic structure; this magnetic order, also called ‘all-in all-out’ ground state, as spins on a tetrahedron point either all in or all out of successive tetrahedra, is pictured in Fig. 1 ▸(*a*). This magnetic order is associated with the one-dimensional IR mΓ_2_^+^ [labelled Γ_3_ in the Kovalev notation used by Lhotel *et al.* (2015 ▸) and Xu *et al.* (2015 ▸)].

The full decomposition in terms of IRs for Wyckoff position 16*d* of 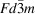 and propagation vector **k** = (0, 0, 0) is 1mΓ_2_^+^(1) ⊕ 2mΓ_4_^+^(3) ⊕ 1mΓ_5_^+^(3) ⊕ 1mΓ_3_^+^(2) (the dimension of each representation is given in brackets).

Thanks to freely available computational tools like *ISODISTORT* (Stokes *et al.*, 2024 ▸), *k-Subgroupsmag* (Perez-Mato *et al.*, 2015 ▸) or *JANA2020* (Petříček *et al.*, 2023 ▸), it is now easy to find the isotropy subgroup (Stokes & Hatch, 1988 ▸) corresponding to any magnetic irreducible representation, so the analysis of a magnetic structure does not have to be restricted only to its relevant IR(s), and in most cases will benefit from the identification of the appropriate MSG (Petříček *et al.*, 2010 ▸). Note that isotropy subgroups can also be called epikernels (Ascher, 1977 ▸), or kernels, for subgroups of minimal symmetry.

**Figure 2:**
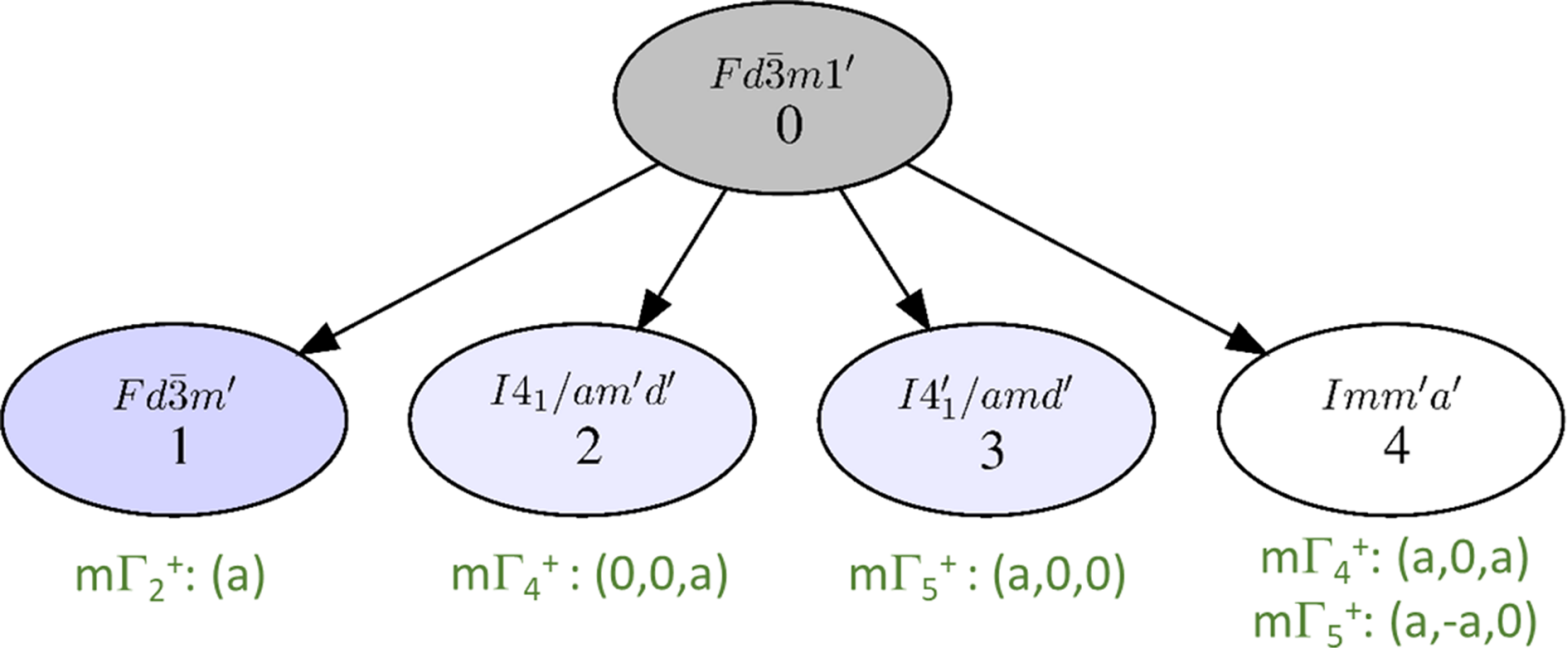
Graph of the maximal subgroups of 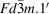 for a propagation vector **k** = (0, 0, 0), allowing a non-zero magnetic moment on Wyckoff position 16*d*. The corresponding IRs are indicated in green, along with their order parameters (see text). For model 4, either mΓ_4_^+^ or mΓ_5_^+^ can result in MSG *Imm*′*a*′, mixing of both IRs is not necessary.

Using *k-Subgroupsmag* for instance, and limiting the subgroup search to maximal subgroups for simplicity, one gets the list of the subgroups of the paramagnetic group 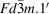 illustrated in Fig. 2 ▸. Time reversal is explicitly included here to avoid confusion with the 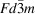 space group, in which time-reversal symmetry operations are not considered, and therefore, cannot be broken.

The correspondence between IRs and MSGs is achieved with *Get_mirreps*. The simplest cases are those for which an MSG corresponds to a single IR of one dimension. In such cases, all symmetry operations of the parent space group are kept, either alone or combined with time reversal. In the example in Fig. 2 ▸, the subgroup of highest symmetry, 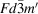, corresponds indeed to the one-dimensional IR mΓ_2_^+^, and is the MSG of the well known all-in all-out magnetic order.

The important elements that have to be given to report this magnetic structure using its MSG are listed in Table 1 ▸. All the information needed is gathered in the magCIF file generated by the symmetry analysis tool used. In this very simple case, filling all this information is rather intuitive, as the unit cell of the magnetic structure and the parent unit cell are the same, and the MSG is in its standard setting. As a result, it is not immediately obvious why all this information is needed, as it makes the description of a rather simple case more complicated. The reason is that, as with any standard, it should be valid for the report of the simplest to the more complex magnetic structure; for the latter, the reason why some items are mandatory will become clearer in the next example.

Let us have a look now at what happens for another type of magnetic ordering observed in pyrochlores, the ‘two-in two-out’ case, exhibited for instance in Tb_2_Sn_2_O_7_ below 0.9 K (Mirebeau *et al.*, 2005 ▸). It also has **k** = (0, 0, 0) order, so the magnetic subgroup should be one of those listed in Fig. 2 ▸ (assuming for simplicity that it is a **k**-maximal subgroup, which is true in this case). In the literature, this order is described with the three-dimensional IR mΓ_4_^+^, so from Fig. 2 ▸, it is clear that the appropriate MSG is *I*4_1_/*am*′*d*′. One can notice immediately that the symmetry of the magnetic order is now lower.

Following the same procedure as done previously (retrieving the information from the magCIF file generated by the computer tool used), Table 2 ▸ can be filled in. In this case, however, several issues need to be pointed out.

In Table 2.6 ▸, the transformation to the standard setting of the MSG is now (½**a** + ½**b**, −½**a** + ½**b**, **c**; ¼, 0, ¼). This is a crucial point: the MSG setting used *is not standard*. This actually means that the list of symmetry operators are *different* from those listed as standard for 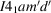 in available *Magnetic Group Tables* (Litvin, 2013 ▸). To be specific, in the online database of MSGs called MGENPOS of the BCS, 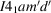 has only 32 symmetry operators (hence 32 as general multiplicity), and it is, not surprisingly, *I* centred. In the setting described here, which is that of the parent structure, there are 16 × 4 = 64 symmetry operations (Tables 2.9 ▸–2.10 ▸), and it is clearly *F* centred (Table 2.10 ▸), as the operations are just a subgroup of the operations of the *F*-centred parent grey group and they are described in the same basis. The symmetry group is however of the type *I*4_1_/*am*′*d*′, because one can choose a different unit cell and origin, where the symmetry operations acquire the form taken as standard for this group type. This is the reason why, in order to define properly an MSG, the transformation to the standard setting should always accompany the MSG label, if it is not in its standard setting; the description of the magnetic symmetry of the structure remains incomplete or ambiguous if only the MSG label is given. Note that this transformation also includes an origin shift (in this case: ¼**a** + ¼**c**, see Table 2.6 ▸).

So why use a non-standard setting at all? Using a non-standard setting is in most cases more convenient, as it allows one to preserve a simple relationship between the parent crystal structure unit cell in the paramagnetic state, and the unit cell of the magnetic structure: in Table 2.2 ▸, one sees that, using this non-standard setting of the MSG, the relationship between the unit cells is still (**a**, **b**, **c**; 0, 0, 0), like in Table 1.2 ▸.

To make this clearer, in the magCIF file, this information is given under:

_parent_space_group.child_transform_Pp_abc ‘a,b,c;0,0,0’

_space_group_magn.transform_BNS_Pp_abc ‘1/2a+1/2b,-1/2a+1/2b,c;1/4,0,1/4’

As explained above, _parent_space_group.child_transform_Pp_abc describes the relationship between the basis (unit cell and origin) of the parent structure and the basis that is being used to describe the magnetic structure. _space_group_magn.transform_BNS_Pp_abc shows a transformation of the basis used to a new basis where the symmetry operations of the MSG would acquire its standard form.

The guidelines strongly advise that the symmetry and symmetry centering operations of the MSG be listed in the setting used. This is the reason why in Table 2 ▸, they are listed as mandatory items (Tables 2.9 ▸–2.10 ▸) along with Tables 2.11–2.13 ▸. Another possible way to describe this magnetic structure would be to use the symmetry operations of the MSG in its standard setting, to which the inverse operation of the transformation (Table 2.6 ▸) is applied. In this case the mandatory items would be Tables 2.4 ▸, 2.6 ▸, 2.8 ▸ and 2.11 ▸–2.13 ▸). Some of the information in Table 2 ▸ is therefore redundant: a certain level of redundancy is recommended in complex cases like this to avoid ambiguities and mistakes, all the more so as the list of symmetry operations is readily available from the magCIF file.

With respect to Tables 2.11 ▸ and 2.12 ▸, one can see that the position of the Tb atom is not split in the subgroup (Table 2.11 ▸), but that the O2 site has split into two orbits, O2_1 and O2_2 (Table 2.12 ▸). A separate description of the non-magnetic atoms in the parent space group is always tempting, especially if there is no structural distortion noticeable, but it is not recommended, as it makes it more difficult to describe the magnetic structure as a single phase, including both atoms and spins. This is the reason why, even if the non-magnetic (split) atoms keep the positions they have in the parent structure, they should still be listed in the magnetic structure report.

Another significant feature of the MSG description is the symmetry constraints on the magnetic moment components (see Table 2.13 ▸). It shows explicitly the degrees of freedom of the moment for each specific site, dictated by the MSG. Additional constraints imposed during the refinement by the user should not appear here; generally, they will be indirectly reflected in the moment component values.

Table 2 ▸ is self-consistent and provides all the information that is necessary to describe the magnetic structure of Tb_2_Sn_2_O_7_. As suggested in the guidelines, information on the active irreducible representation can also be given for completeness (Table 2.14 ▸). In complex cases involving several possible IRs, it can be very useful, for a better understanding of the phase transition for instance, as will be illustrated in later examples.

These two simple examples underline the key points of a comprehensive report of a magnetic structure using its MSG. Both structures are from the simplest and most frequent case mentioned in the guidelines, where the MSG of the structure is only compatible with a single IR. They show how one can easily deduce the MSG knowing the IR involved, and vice-versa, using available magnetic symmetry computer tools. As an additional note, the active IR, mΓ_4_^+^, of the magnetic structure of Tb_2_Sn_2_O_7_, is three dimensional. This means that for this IR, several different MSGs are possible, depending on the order parameters direction in the IR space, that is, depending on the combination of the basis modes. In the particular case of mΓ_4_^+^ with special direction (0,0,*a*), the MSG of maximal symmetry 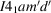 is realized, but different combinations of the basis modes could lead to different MSGs, as illustrated in Fig. 2 ▸ with mΓ_4_^+^: (*a*,0,*a*), which leads to another maximal subgroup, *Imm*′*a*′.

#### 2.1.2. Single k = (½, 0, 0) magnetic order in BiMnTeO_6_

BiMnTeO_6_ has a monoclinic *P*2_1_/*c* crystal structure. Mn spins (Wyckoff position 4*e*, general multiplicity) order below *T*_N_ = 10 K, with propagation vector **k** = (½, 0, 0) (Matsubara *et al.*, 2019 ▸). In Matsubara *et al.* (2019 ▸), magnetic order is determined using representation theory. There are four irreducible representations of one dimension, each contained three times (three basis vectors), according to the decomposition 3mY_1_^+^(1) ⊕ 3mY_1_^−^(1) ⊕ 3mY_2_^+^(1) ⊕ 3mY_2_^−^(1).

**Figure 3:**
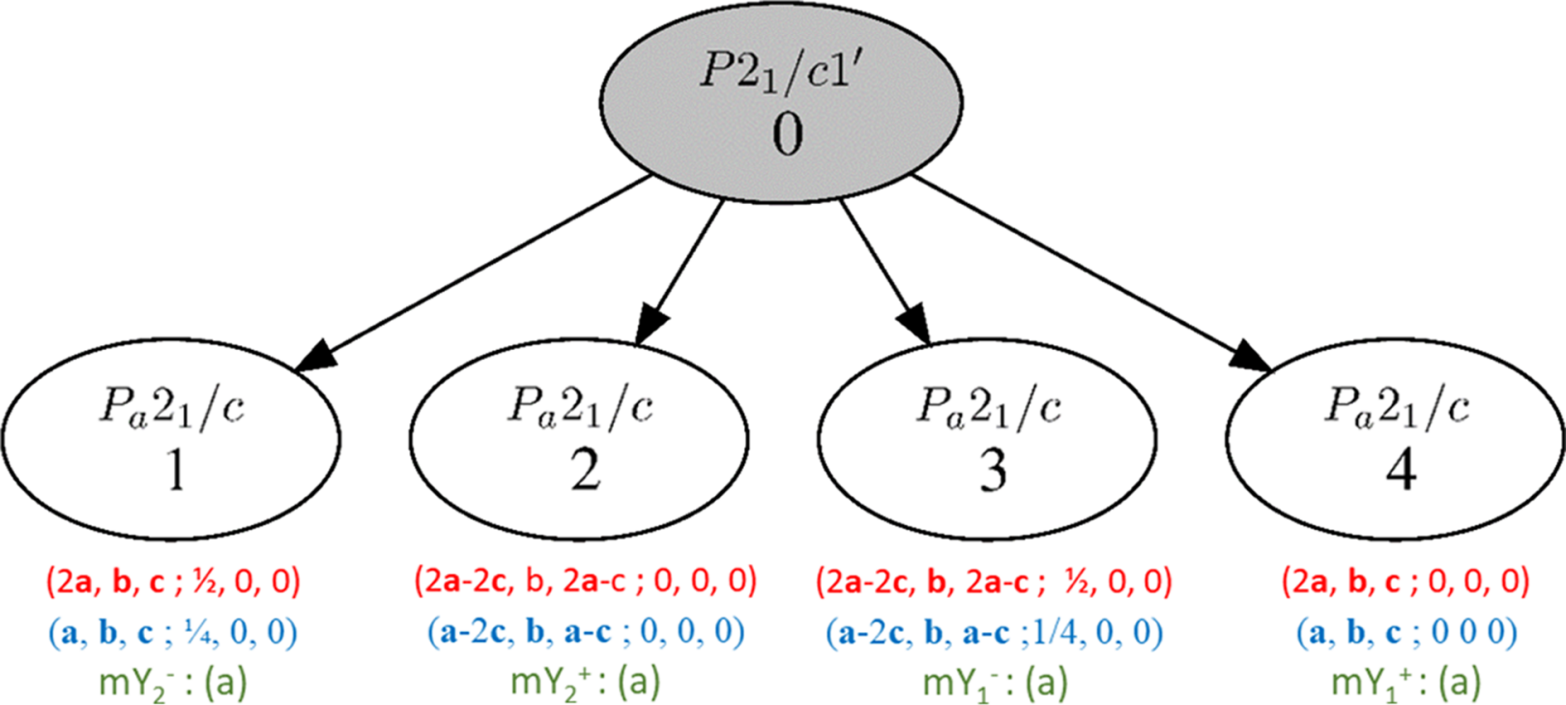
Graph of the maximal subgroups of *P*2_1_/*c*.1′ for the propagation vector **k** = (½, 0, 0), allowing a non-zero magnetic moment on Wyckoff position 4*e*. The corresponding IRs are indicated in green (see text). The transformation from the parent unit cell to the standard setting of the MSG type is shown in red, in blue the transformation corresponding to the magCIF tag: _space_group_magn.transform_BNS_Pp_abc, which is the transformation to the standard setting of the MSG type, not of the parent unit cell, but of the unit cell chosen for the description of the magnetic structure. In the basis of the parent unit cell, the magnetic unit cell is related with the parent one following (2**a**, **b**, **c**; 0, 0, 0), because of the propagation vector (**k** = ½, 0, 0).

In terms of MSGs, one gets the graph illustrated in Fig. 3 ▸.

From Fig. 3 ▸, each irreducible representation leads to a magnetic order which can be described with the same magnetic space-group type *P_a_*2_1_/*c* (subscript *a* corresponds to the anti-translation {1′ | ½, 0, 0}). However, these four magnetic space groups are different, as will be explained below. This is an obvious example in which reporting the magnetic space group label and the magnetic moment values on the Mn sites only is clearly not enough for a full description of the magnetic ordering.

In fact, each of the four magnetic structures that can be derived from Fig. 3 ▸ have an MSG of type *P_a_*2_1_/*c*, but these four groups are different non-equivalent subgroups of the parent grey group. They are formed by different subsets of symmetry operations, when described in the parent basis. As a consequence, different changes of unit cell and origin are required to transform these symmetry operations to their standard form for the MSG *P_a_*2_1_/*c*.

**Figure 4:**
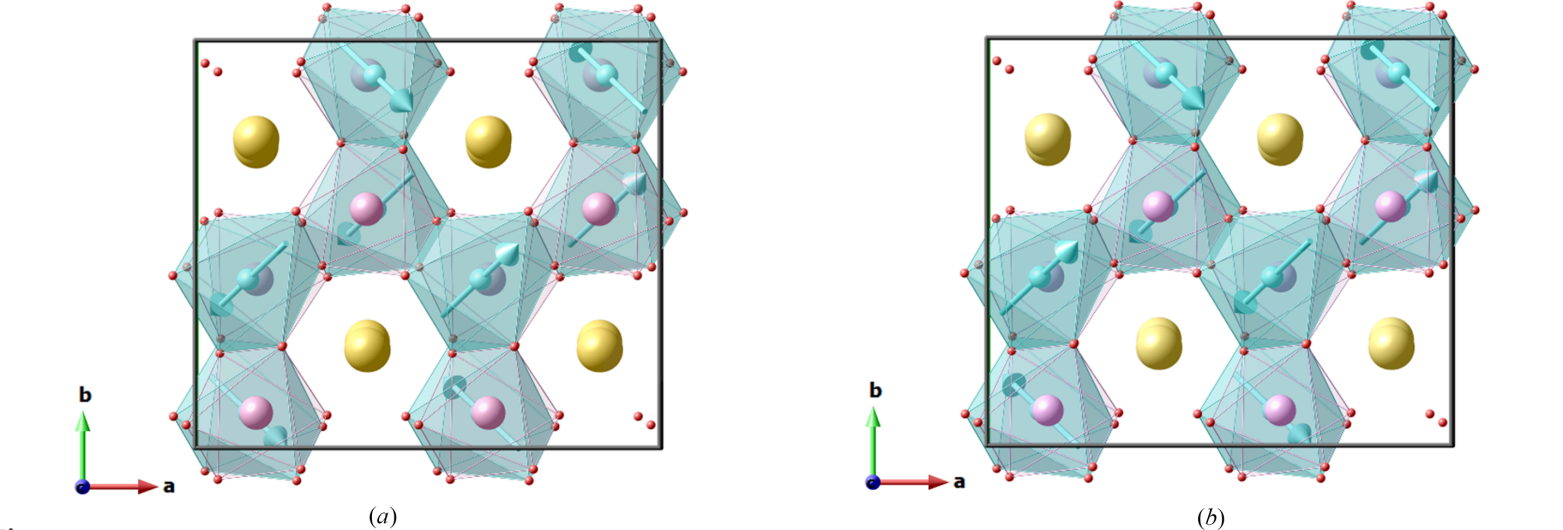
Possible spin arrangements for BiMnTeO_6_, corresponding to model 1 (*a*) and model 4 (*b*) (see text). Mn atoms are shown as light blue, Bi, Te and O atoms are pictured as yellow, pink and red spheres. The Mn spin components are from Matsubara *et al.* (2019 ▸). BiMnTeO_6_ orders below 10 K according to model 1 (Matsubara *et al.*, 2019 ▸).

As an example, a comparison between model 1 with subgroup *P_a_*2_1_/*c* (2**a**, **b**, **c**; ½, 0, 0) and model 4 with subgroup *P_a_*2_1_/*c* (2**a**, **b**, **c**; 0, 0, 0) is instructive (see Fig. 4 ▸). If both models are described using a supercell (2**a**, **b**, **c**; 0, 0, 0), without changing the origin with respect to the parent structure, the first model would still require a shift of the origin by (½, 0, 0) of the magnetic supercell to acquire the standard form of the MSG *P_a_*2_1_/*c*. This means that different symmetry operations are kept in the two models. For instance, model 4 has the symmetry operations {−1| 0 0 0} and {−1′| ½ 0 0}, which have the standard form expected in the MSG *P_a_*2_1_/*c*, while in model 1 the operations are {−1| ½ 0 0} and {−1′| 0 0 0}. One can see from Table 3 ▸ and Fig. 4 ▸ that this has significant consequences on the symmetry dictated relations between the magnetic moment components of symmetry related atoms.

A note on the MSG notation: in this example, the MSG notation varies between the two possible standards, BNS or OG, as the **k** = (½, 0, 0) propagation vector implies an anti-translation (translation associated with time reversal), and thus a type IV MSG. The BNS notation is *P_a_*2_1_/*c*, as already mentioned, with the subscript *a* indicating the anti-translation along **a**. In the OG notation, this is *P*_2*a*_2_1_/*c*. In the newly defined UNI standard, this MSG is written *P*2_1_/*c*.1′*_a_* [*P*2_1_/*c*]. This notation includes the time-reversal operator explicitly, following the other point operation symbols, so that it is straightforward to deduce that the magnetic point group is 2/*m*.1′, that is, a grey point group, as for all type IV MSGs. The subscript identifying the anti-translation is written on the time-reversal generator symbol (1′*_a_*). Inside the square brackets is indicated information about the family space group (*i.e.* the non-magnetic space group obtained by removing time reversal from each time-reversed symmetry operation). In this case it does not add any important information and the truncated form *P*2_1_/*c*.1′*_a_* can be used as an alternative.

Table 4 ▸ illustrates how to report the magnetic ordering of BiMnTeO_6_ under its MSG. There is no additional difficulty with respect to the previous example.

#### 2.1.3. Single **k** = **(⅓, ⅓, 0)** in hexagonal **BaMnO_3_(2H)**

**Figure 5:**
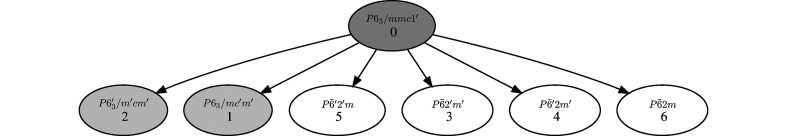
Graph of the maximal subgroups of *P*6_3_/*mmc.*1′ for the propagation vector **k** = 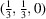 (whole star), allowing a non-zero magnetic moment on Wyckoff position 2*a*.

This example is an illustration of a slightly more complex but nevertheless quite common case. BaMnO_3_(2H) is a hexagonal form of BaMnO_3_, which crystallizes in space group *P*6_3_/*mmc* (No. 194). Mn magnetic species sit on Wyckoff position 2*a*(0, 0, 0). Below *T*_N_ = 2.3 K, there is a magnetic ordering transition, characterized by the propagation vector **k** = (⅓, ⅓, 0) (Nørlund Christensen & Ollivier, 1972 ▸). The possible maximal MSGs compatible with this propagation vector are shown in Fig. 5 ▸.

In the spin arrangement reported by Christensen *et al.* (Nørlund Christensen & Ollivier, 1972 ▸), the coupling along **c** is antiferromagnetic, which rules out model 1 (*P*6_3_/*mc*′*m*′, No. 193.260), since it only allows ferromagnetic ordering along **c** (magnetic point group 6/*mm*′*m*′). Model 2 (*P*6_3_′/*m*′*cm*′, No. 193.259), on the other hand, provides a perfect match to the model given by Nørlund Christensen & Ollivier (1972 ▸).

Table 5 ▸ can be filled in from the magCIF information provided by the symmetry analysis tools, following the same procedure as before. Note that in Table 5.13 ▸, Mn1_1 and Mn1_2 have been constrained to have the same moment amplitude, but this is not symmetry imposed, as these two atoms sit on two different orbits. The model has clearly two magnetic degrees of freedom (or two modes), associated with the two m_*z*_ components of the split Mn sites.

In order to maintain a more direct visual relation with the parent structure, the unit cell used for this description is a supercell of the parent unit cell, which keeps its orientation, but with **a** and **b** tripled (see Table 5.2 ▸). This is a ninefold supercell, while the actual periodicity of the structure can be generated by a smaller threefold supercell, as indicated by the transformation to standard [(⅓**a** − ⅓**b**, ⅓**a** + ⅔**b**, **c**; 0, ⅓, 0), see Table 5.6 ▸]. The use of a non-standard larger supercell requires that non-standard centering translations (see Table 5.10 ▸) are included to describe the lattice. In such cases, it can be more advantageous to describe the magnetic ordering in the standard setting of its MSG, an operation which can be easily performed on the BCS, as the user is always free to choose any alternative setting deemed appropriate.

**Figure 6:**
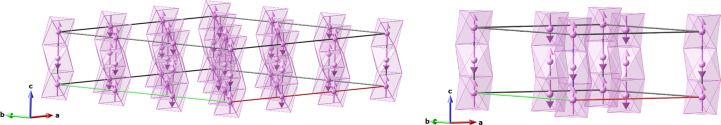
Magnetic order of BaMnO_3_(2H), described in two different unit cells: (*a*) parent cell setting and (*b*) standard setting of *P*6_3_/*m*′*cm*′ (see also Tables 5 ▸ and 6 ▸). Only magnetic atoms (Mn, in pink) are represented, inside their oxygen octahedral environment.

The resulting description in the standard setting of the MSG is given in Table 6 ▸, which is absolutely equivalent to the description using the parent-like unit-cell setting of Table 5 ▸. In this case the transformation to standard setting in Table 6.6 ▸ becomes (**a**, **b**, **c**; 0, 0, 0), as expected since a standard setting is used, and the magnetic unit cell is three times smaller (compare Table 5.8 ▸ and Table 6.8 ▸, see also Fig. 6 ▸). On the downside, the relationship between the parent cell and the unit cell of the magnetic structure becomes more complex to visualize (Table 6.2 ▸).

It is also of interest here to compare the irreducible representation approach with the MSG one. The decomposition of the magnetic representation into IRs for the **k** = (⅓, ⅓, 0) ordering of BaMnO_3_(2H) is mK_3_(1) ⊕ mK_4_(1) ⊕ mK_5_(2) ⊕ mK_6_(2) (Mn on Wyckoff position 2*a*). From the basis functions (obtained with BasIreps in this example, see Table 7 ▸) of the two one-dimensional representations mK_3_ and mK_4_, one can see that mK_4_ corresponds to a parallel arrangement of the Mn spins along **c**, which is, as mentioned earlier, not compatible with the model proposed by Christensen *et al.* (Nørlund Christensen & Ollivier, 1972 ▸), while mK_3_ corresponds to an antiparallel coupling along **c**, which agrees with that model.

Using a description of a magnetic structure based on a single basis vector will lead however to an amplitude modulated spin on the Mn site, because of the (⅓, ⅓, 0) propagation vector. Depending on the phase of the modulation, this can lead for instance to a up-down-down magnetic ordering, with m_*z*_(Mn1_1) = −m_*z*_(Mn1_2)/2. Such a modulation of the moment amplitude is not imposed by the MSG description, which leaves m_*z*_(Mn1_1) and m_*z*_(Mn1_2) independent, as can be seen from the constraints of Table 5.13 ▸ or Table 6.13 ▸. Besides, the published model (Fig. 6 ▸) constrained m_*z*_(Mn1_1) = −m_*z*_(Mn1_2) = 3 μ_B_.

**Figure 7:**
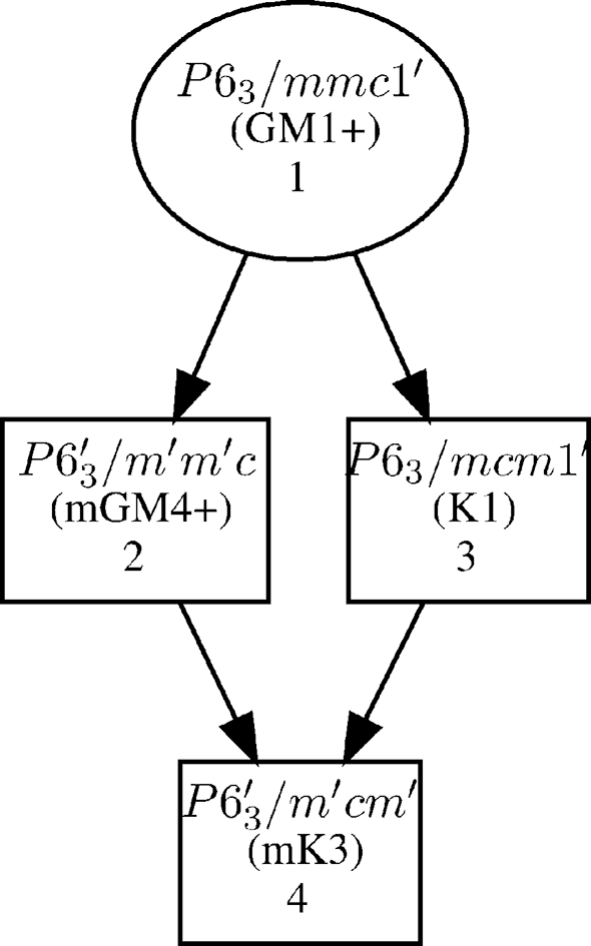
Graph of intermediate subgroups for the *P*6_3_/*mmc*.1′ → *P*6_3_/*m*′*cm*′ group–subgroup pair.

To better understand this discrepancy between the two approaches, it is useful to map for the parent group-magnetic subgroup pair the list of compatible IRs; this can be achieved for instance with the *Get_mirreps* tool of the BCS, which provides, along with the compatible IRs, the direction within the IR space, and the corresponding isotropy subgroup. For the *P*6_3_/*mmc*.1′ *→ P*6_3_/*m*′*cm*′ group–subgroup pair of this example, one gets the graph shown in Fig. 7 ▸.

This graph shows explicitly that, in addition to the primary IR mK_3_, which is responsible for the magnetic ordering at the K point (⅓, ⅓, 0), there is a secondary IR, mΓ_4_^+^, corresponding to the propagation vector **k** = (0, 0, 0), which is also symmetry compatible with the *P*6_3_/*m′cm′* group. This propagation vector actually corresponds to the third harmonic of the **k** = (⅓, ⅓, 0) primary order. Using an MSG approach in this case thus automatically includes this secondary magnetic degree of freedom. This is actually a general feature of using MSGs: all magnetic degrees of freedom corresponding to secondary IRs, which are symmetry allowed, are included. Most of the time they can be neglected and do not really increase the number of degrees of freedom (Gallego *et al.*, 2016 ▸). In the BaMnO_3_(2H) case, this additional degree of freedom leads to independent moments on the Mn1_1 and Mn1_2 sites: the collinear up-down-down model with all moments having the same amplitude requires the presence of the secondary IR mΓ_4_^+^[**k** = (0, 0, 0)] in addition to the primary IR mK_3_. The mode mK_3_ on its own would lead to Mn1_1 and Mn1_2 moments constrained to m_*z*_(Mn1_2) = −m_*z*_(Mn1_1)/2 (Table 8 ▸). Note that the presence of a **k** = (0, 0, 0) component implies that magnetic intensity will superpose to structural Bragg peaks; experimentally, the detection of the presence or not of this secondary IR should therefore be quite straightforward. As harmonics of a primary propagation vector are not independent of the latter, the magnetic arrangement is strictly considered a 1-**k** magnetic structure.

Following the guidelines, an additional table containing all the information on the IRs and their modes can be added to the description of the magnetic structure, as shown in Table 8 ▸.

A note about Table 8.1 ▸: the dimension of the full IR is two, that is, twice the dimension of the small IR (mK_3_ being one dimensional, see the IR decomposition above). This is because the propagation vectors **k** and −**k** are not equivalent. In the case of a single-**k** structure, in which **k** and −**k** are equivalent, the dimension of the full IR is that of the small IR, as only one propagation vector is involved.

### 2.2. Multi-**k** magnetic structure in TbCrO_3_

Multi-**k** structures can be handled just as easily as single-**k** ones with MSGs. This is illustrated in the following with the case of TbCrO_3_, a distorted perovskite (*Pnma*, No. 62, *a* = 5.513 Å, *b* = 7.557 Å, *c* = 5.291 Å), with two independent magnetic orderings (Bertaut *et al.*, 1967 ▸). Below *T*_N1_ = 158 K, Cr spins (Wyckoff position 4*b*) order with propagation vector **k** = (0, 0, 0); below 4 K (*T*_N2_ not defined precisely) Tb spins (4*c* position) also partially order with the same propagation vector. Below *T*_N3_ = 3.05 K, a new Tb spins order is observed, with propagation vector **k** = (½, 0, 0), thus further lowering the symmetry.

**Figure 8:**
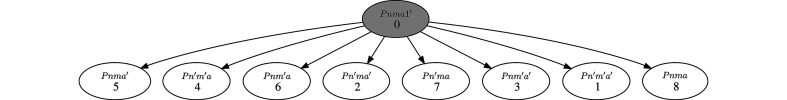
Graph of the maximal subgroups of *Pnma*.1′ for the propagation vector **k** = (0, 0, 0). Restricting the analysis to those space groups allowing a non-zero moment on Wyckoff position 4*b* (Cr) further reduces the possibilities to *Pn*′*ma*′, *Pnm*′*a*′, *Pn*′*m*′*a* and *Pnma*.

The first magnetic transition can be described by the graph in Fig. 8 ▸. There are eight possible MSGs, all **k**-maximal. MSG *Pn*′*m*′*a*, No. 62.446 (model 4) fits the description of the TbCrO_3_ magnetic structure given by Bertaut *et al.* (1967 ▸), corresponding to a G-type ordering of the Cr magnetic moments along **b**, and a possible A-type ordering of the Tb moments along **a** (Wollan & Koehler, 1955 ▸). In terms of IRs, the decomposition leads to eight possible magnetic representations, all one dimensional; the active representation corresponding to *Pn*′*m*′*a* is mΓ_2_^+^.

**Figure 9:**
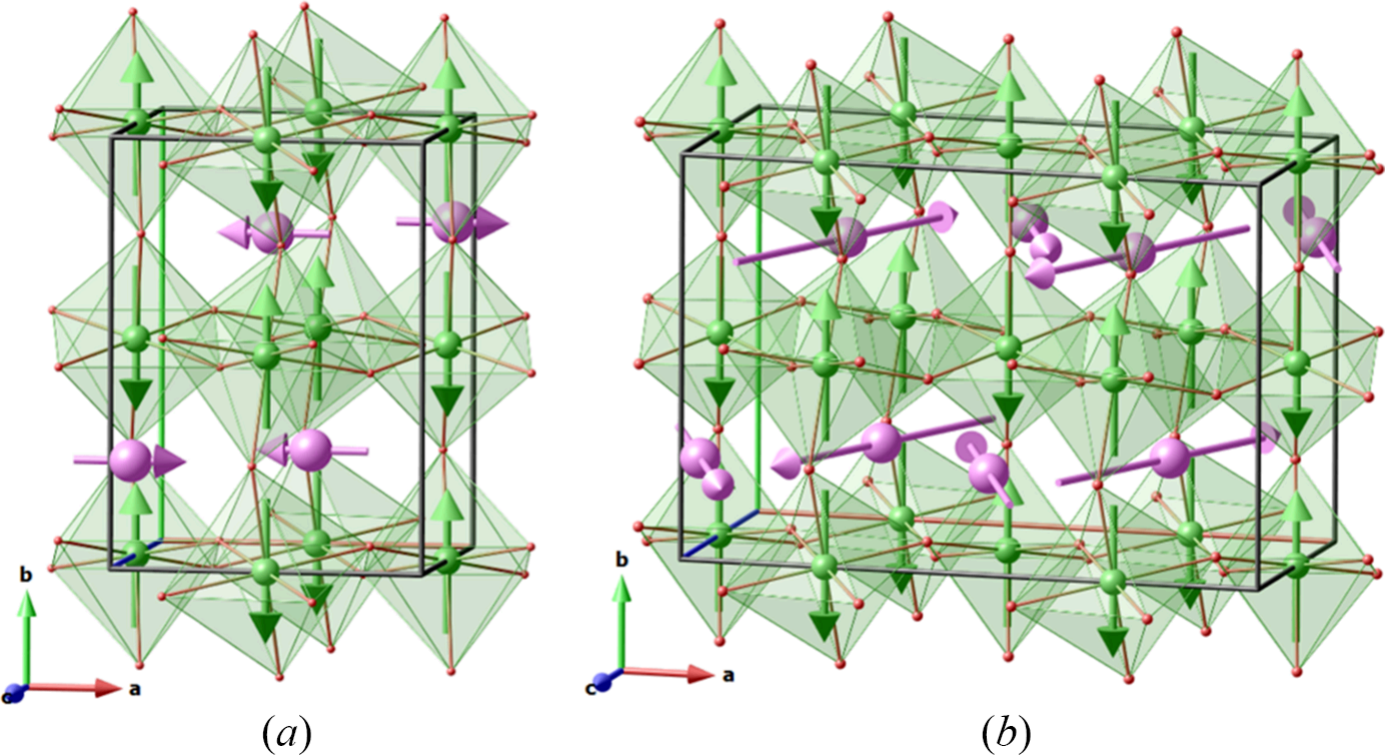
(*a*) Magnetic structure of TbCrO_3_ (*T*_N3_ < *T* < *T*_N2_). (*b*) Magnetic structure of TbCrO_3_ (*T* < *T*_N3_). Cr atoms and spins in green, Tb atoms and spins in purple. CrO_6_ octahedra are also drawn. Spins are not drawn to scale for clarity purposes [from Bertaut *et al.* (1967 ▸)].

The magnetic structure is illustrated in Fig. 9 ▸(*a*) and described in Table 9 ▸. This first ordering is therefore a standard case of a 1-**k** magnetic ordering, corresponding to the **k**-maximal subgroup *Pn*′*m*′*a*, with mΓ_2_^+^ as the primary IR. This MSG has five degrees of freedom (Table 9.10 ▸), but only the moment along **b** of the Cr atoms have been refined by Bertaut *et al.* (1967 ▸). Note that because this is a fairly trivial case with the MSG in its standard setting, the transformation to a standard setting and the MSG symmetry operations have been omitted in Table 9 ▸. As a rule, if no transformation is given, it implicitly means that the MSG is in its standard setting.

Below *T*_N3_ = 3.05 K, a new ordering characterized by the propagation vector **k** = (½, 0, 0) is observed in addition to the existing **k** = (0, 0, 0) order.

**Figure 10:**
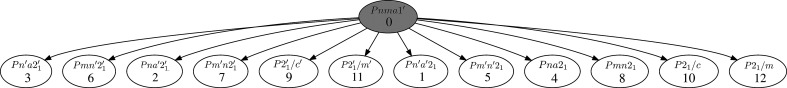
Graph of the subgroups of *Pnma*.1′ allowing a non-zero magnetic moment on the Wyckoff positions 4*b* and 4*c* for two propagation vectors **k** = (0, 0, 0) and **k** = (½, 0, 0).

This corresponds to an additional symmetry breaking, which can be studied using the *k-Subgroupsmag* tool, as the latter offers the possibility to perform a group–subgroup analysis with several propagation vectors. The result is shown in Fig. 10 ▸ (only MSGs allowing a non-zero magnetic moment on both Cr and Tb sites have been considered). There are now 12 possible **k**-maximal MSGs.

**Figure 11:**
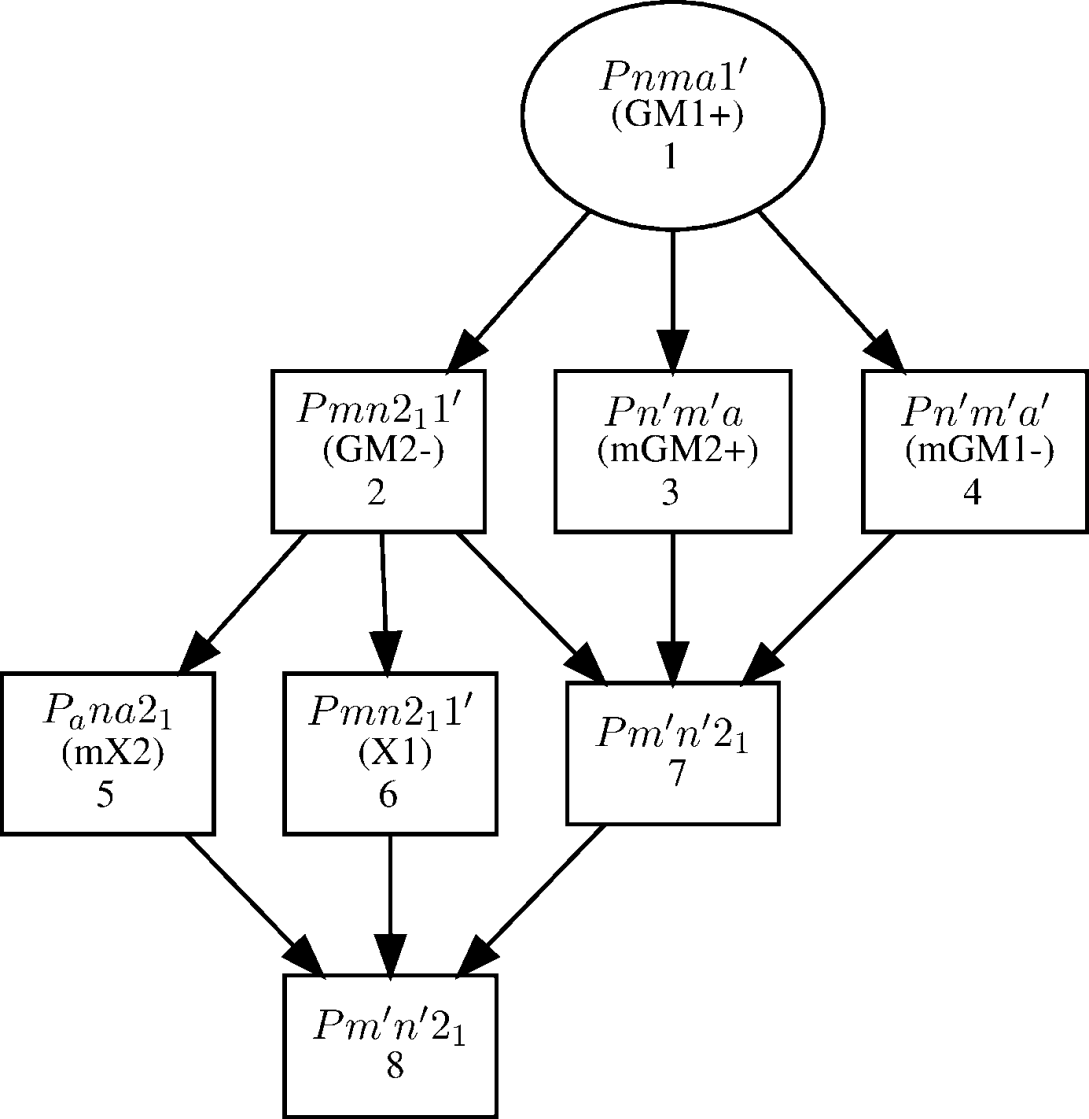
Graph of the isotropy subgroups for the *Pnma*.1′ → *Pm*′*n*′2_1_ group–subgroup pair.

The IR decomposition which can be done in parallel leads to two more possible representations of dimension 2, mX_1_ and mX_2_, for either the Wyckoff position 4*b* or 4*c* and **k** = (½, 0, 0), in addition to the eight possible one-dimensional IRs corresponding to **k** = (0, 0, 0). The symmetry constraints given by Bertaut *et al.* (1967 ▸) for the Tb ordering correspond to MSG *Pm*′*n*′2_1_ (No. 31.127) in the graph shown in Fig. 10 ▸. The corresponding group–subgroup hierarchy is illustrated in Fig. 11 ▸.

From this graph, one can see that there are two primary IRs, mΓ_2_^+^ and mX_2_, which means that, according to the Landau theory of phase transition, there are two order parameters: those correspond comprehensibly to the ordering of the Cr and Tb spins. IR mΓ_1_^−^ is allowed as a secondary mode. It does not affect the ordering of the Cr spins, but could potentially be involved in a more complex model, in which the amplitude of the Tb moments are not equal. However, in the model proposed by Bertaut *et al.* (1967 ▸), all Tb have equal ordered moment values, implying that this mode has zero amplitude.

The MSG description of the 2-**k** magnetic ordering of TbCrO_3_ below *T*_N3_ is detailed in Table 10 ▸. Table 10.13 ▸ indicates that there are up to 14 magnetic degrees of freedom involved in this magnetic transition. In fairly complex cases like this, it can be advantageous therefore to decompose the MSG in terms of basis modes, using *ISODISTORT*. In this example, the basis modes of mX_2_ and mΓ_2_^+^ show that the 14 degrees of freedom allowed by *Pm*′*n*′2_1_ are divided into seven for mX_2_ (four basis modes for Tb and three for Cr), five for mΓ_2_^+^ (two for Tb and three for Cr), and two for mΓ_1_^−^ (for Tb spins only). If one adds the condition that Tb moments only are involved in IR mX_2_, and similarly, Cr moments only are involved in IR mΓ_2_^+^, the list of modes in Table 11.2 ▸ is limited to seven modes in total, thus reducing to seven the number of degrees of freedom (Table 11 ▸). Like in the published model, one can also impose an equal amplitude of the moment components for the same magnetic species, thus further reducing the number of freedom to five (that is, m_*x*_, m_*y*_, m_*z*_ for Cr and m_*x*_ and m_*z*_ for Tb). Practically speaking, refining the amplitudes (C_*i*_) of the modes can be directly performed by *FullProf* using the adequate option: in this instance, five modes only need to be refined, as for a constant Tb moment one cannot have both modes one and two, or both modes three and four, active together (Table 11.2 ▸).

This is an example where, as several IRs are compatible with the MSG, the restriction to a single primary IR for Tb (excluding mΓ_1_^−^) introduces additional constraints, which are not taken into account by the MSG: using the MSG description with its 14 degrees of freedom would be quite inefficient. As these restrictions are valuable for the determination of the magnetic structure, they are therefore useful to indicate, as shown in Table 11 ▸. As mentioned in the guidelines, this step is not compulsory, however, as, in practice, it requires caution. In most cases, the choice of the asymmetric units can differ depending on the computing tool that is used: as a result, to make sure that the listing of atoms and of basis modes correspond to the same description and are consistent with each other, one often has to use alternate settings or origin changes, which can be a source of mistakes.

In this more complex case of an MSG use, one should keep in mind that one of the strong points of MSGs is the fact that their magnetic point groups can be quickly and easily inferred, thus giving information on the physical properties of the system under study. In this example, from Table 10.7 ▸, one can deduce that TbCrO_3_ is polar along **c**, and allows ferromagnetism along **c** as well.

## 3. Conclusion

With the recent development of a variety of computational tools, it is now possible to apply magnetic symmetry to the understanding of a magnetic structure, rather simply and methodically. The standard magCIF format that has been implemented in most of these tools now allows one to communicate a magnetic structure in a standard way, in a similar way to the CIF format which is now widely used in the crystallography community. *Guidelines for communicating commensurate magnetic structures* (Perez-Mato *et al.*, 2024 ▸) describes how to report a magnetic structure in a standard and non-ambiguous way. The four examples treated in this article apply the guidelines to cases that are likely to be encountered by any researcher working in the field of magnetic compounds. It explains specific key points of the guidelines for a better understanding of the important information that is needed. These examples underline in parallel a few advantages of using the magnetic space group approach, or a combination of both magnetic space groups and irreducible representations, when reporting a magnetic structure. Beyond the purely mathematical description, MSGs provide useful insights on the physics behind a magnetic ordering transition.

## Figures and Tables

**Table 1:** Description of the magnetic structure of Nd_2_Zr_2_O_7_ under its MSG [converted from the model reported by Xu *et al.* (2015 ▸)] The model reported by Lhotel *et al.* (2015 ▸) differs by the amplitude of the ordered moment, |*M*| = 0.8 μ_B_. Example of mandatory information in brown.

		Nd_2_Zr_2_O_7_ magnetic structure (MAGNDATA, No. 0.340)						
1.1	Parent space group	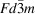 (No. 227)						
1.2	Transformation from parent basis to magnetic structure unit cell	(**a**, **b**, **c**; 0, 0, 0)						
1.3	Propagation vector	**k** = (0, 0, 0)						
1.4–1.5	MSG symbol and number	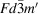 and 227.131						
1.6	Transformation to standard setting of MSG	(**a**, **b**, **c**; 0, 0, 0)						
1.7	Magnetic point group	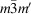 (32.4.121)						
1.8	Magnetic unit-cell parameters (Å, °)	**a** = **b** = **c** = 10.6611						
		α = β = γ = 90						
1.9	MSG symmetry operations (48)	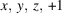			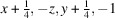			
		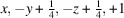			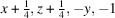			
		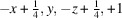			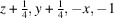			
		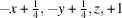			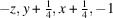			
		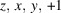			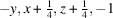			
		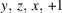			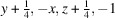			
		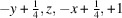			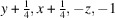			
		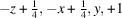			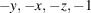			
		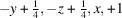			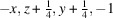			
		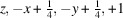			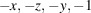			
		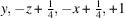			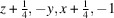			
		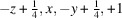			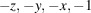			
		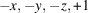			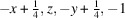			
		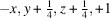			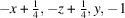			
		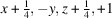			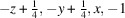			
		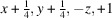			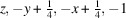			
		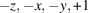			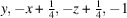			
		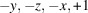			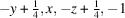			
		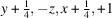			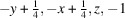			
		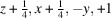			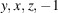			
		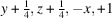			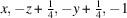			
		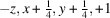			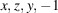			
		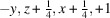			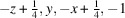			
		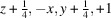			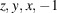			
1.10	MSG symmetry centering operations (4)	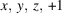						
		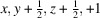						
		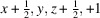						
		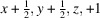						
1.11	Positions (label, *x*, *y*, *z*) of magnetic atoms (1)	Nd	0.5		0.5		0.5	
1.12	Positions (label, *x*, *y*, *z*) of non-magnetic atoms (3)	Zr	0		0		0	
		O1	0.375		0.375		0.375	
		O2	0.3357		0.125		0.125	
1.13	Magnetic moment components (m_*x*_, m_*y*_, m_*z*_), symmetry constraints and moment amplitude (|M|, in μ_B_) (1)	Nd	0.73	0.73	0.73	m_*x*_, m_*x*_, m_*x*_		1.26

**Table 2:** Description of the magnetic structure of Tb_2_Sn_2_O_7_ under its MSG [converted from the model reported by Mirebeau *et al.* (2005 ▸)] Example of mandatory information in brown.

		Tb_2_Sn_2_O_7_ magnetic structure (MAGNDATA, No. 0.48)						
2.1	Parent space group	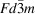 (No. 227)						
2.2	Transformation from parent basis to magnetic structure unit cell	(**a**, **b**, **c**; 0, 0, 0)						
2.3	Propagation vector	**k** = (0, 0, 0)						
2.4–2.5	MSG symbol and number	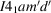 and 141.557						
2.6	Transformation to standard setting of MSG	(½**a** + ½**b**, −½**a** + ½**b**, **c**; ¼, 0, ¼)						
2.7	Magnetic point group	4/*mm*′*m*′ (15.6.58)						
2.8	Magnetic unit-cell parameters (Å, °)	**a** = **b** = **c** = 10.426						
		α = β = γ = 90						
2.9	MSG symmetry operations (16)	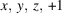			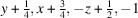			
		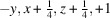			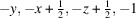			
		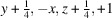			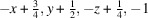			
		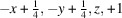			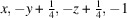			
		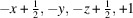			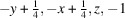			
		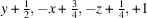			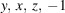			
		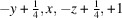			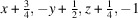			
		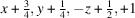			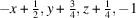			
2.10	MSG symmetry centering operations (4)	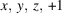						
		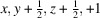						
		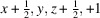						
		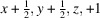						
2.11	Positions (label, *x*, *y*, *z*) of magnetic atoms (1)	Tb	0.5		0.5		0.5	
2.12	Positions (label, *x*, *y*, *z*) of non-magnetic atoms (4)	Sn	0		0		0	
		O1	0.375		0.375		0.375	
		O2_1	0.336		0.125		0.125	
		O2_2	0.125		0.125		0.336	
2.13	Magnetic atom, moment components (m_*x*_, m_*y*_, m_*z*_), symmetry constraints and moment amplitude (|M|, in μ_B_) (1)	Tb	3.85	3.85	2.80	m_*x*_, m_*x*_, m_*z*_		6.1
2.14	Primary IR (dimension)	mΓ_4_^+^ (three-dimensional) (special direction)						

**Table 3:** Details of the magnetic structure models 1 and 4 (obtained with *MAGMODELIZE*) for Mn on Wyckoff position 4*e* Opposite moment components are written in red to underline the differences between the two models. See Fig. 3 ▸ for the correspondence between model number and MSG.

		Multiplicity	Constraints
Model 1	(*x*, *y*, *z* | m_*x*_, m_*y*_, m_*z*_) (−*x*, *y* + 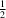 , −*z* + 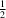 | m_*x*_, −m_*y*_, m_*z*_)	8	(m_*x*_, m_*y*_, m_*z*_)
	(−*x*, −*y*, −*z* | −m_*x*_, −m_*y*_, −m_*z*_) (*x*, −*y* + 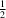 , *z* + 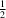 | −m_*x*_, m_*y*_, −m_*z*_)		
	(*x* + 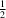 , *y*, *z* | −m_*x*_, −m_*y*_, −m_*z*_) (−*x* + 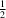 , *y* + 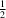 , −*z* + 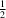 | −m_*x*_, m_*y*_, −m_*z*_)		
	(−*x* + 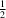 , −*y*, −*z* | m_*x*_, m_*y*_, m_*z*_) (*x* + 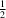 , −*y* + 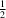 , *z* + 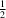 | m_*x*_, −m_*y*_, m_*z*_)		
Model 4	(*x*, *y*, *z* | m_*x*_, m_*y*_, m_*z*_) (−*x*, *y* + 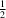 , −*z* + 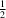 | −m_*x*_, m_*y*_, −m_*z*_)	8	(m_*x*_, m_*y*_, m_*z*_)
	(−*x*, −*y*, −*z* | m_*x*_, m_*y*_, m_*z*_) (*x*, −*y* + 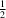 , *z* + 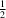 | −m_*x*_, m_*y*_, −m_*z*_)		
	(*x* + 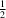 , *y*, *z* | −m_*x*_, −m_*y*_, −m_*z*_) (−*x* + 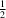 , *y* + 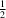 , −*z* + 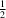 | m_*x*_, −m_*y*_, m_*z*_)		
	(−*x* + 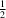 , −*y*, −*z* | −m_*x*_, −m_*y*_, −m_*z*_) (*x* + 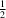 , −*y* + 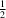 , *z* + 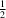 | m_*x*_, −m_*y*_, m_*z*_)		

**Table 4:** Description of the magnetic structure of BiMnTeO_6_ under its MSG [converted from the model reported by Matsubara *et al.* (2019 ▸)] Example of mandatory information in brown.

		BiMnTeO_6_ magnetic structure (MAGNDATA, No. 1.301)					
4.1	Parent space group	*P*2_1_/*c* (No. 14)					
4.2	Transformation from parent basis to magnetic structure unit cell	(2**a**, **b**, **c**; 0, 0, 0)					
4.3	Propagation vector	**k** = (½, 0, 0)					
4.4–4.5	MSG symbol and number	*P_a_*2_1_/*c* and 14.80 (BNS) – *P*2_1_/*c*.1′_a_ (UNI)					
4.6	Transformation to standard setting of MSG	(**a**, **b**, **c**; ¼, 0, 0)					
4.7	Magnetic point group	2/*m*.1′ (5.2.13)					
4.8	Magnetic unit-cell parameters (Å, °)	**a** = 10.3322 (2), **b** = 9.0579 (1), **c** = 9.9033 (1)					
		α = 90, β = 90.162 (2), γ = 90					
4.9	MSG symmetry operations (4)	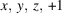					
		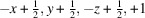					
		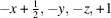					
		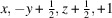					
4.10	MSG symmetry centering operations (2)	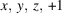					
		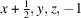					
4.11	Positions of magnetic atoms (label, *x*, *y*, *z*) (1)	Mn	0.38620		0.91340		0.75110
4.12	Positions of non-magnetic atoms (label, *x*, *y*, *z*) (8)	Bi	0.13030		0.75930		0.00000
		Te	0.13110		0.08590		0.73720
		O1	0.31330		0.74840		0.65340
		02	0.45185		0.42270		0.86360
		03	0.44745		0.73220		0.87150
		04	0.22785		0.94760		0.84790
		05	0.48080		0.05640		0.84730
		06	0.21710		0.58040		0.87140
4.13	Magnetic atom, moment components (m_*x*_, m_*y*_, m_*z*_), symmetry constraints and moment amplitude (|M|, in μ_B_) (1)	Mn	1.7 (1)	−1.6 (1)	2.8 (1)	m_*x*_, m_*x*_, m_*z*_	3.7 (2)
4.14	Primary IR (dimension)	mY_2_^−^ (one-dimensional)					

**Table 5:** Description of the magnetic structure of BaMnO_3_(2H) under its magnetic space group – parent-like cell MSG setting Example of mandatory information in brown.

		BaMnO_3_(2H) magnetic structure						
5.1	Parent space group	*P*6_3_/*mmc* (No. 194)						
5.2	Transformation from parent basis to magnetic structure unit cell	(3**a**, 3**b**, **c**; 0, 0, 0)						
5.3	Propagation vector	**k** = (⅓, ⅓, 0)						
5.4–5.5	MSG symbol and number	*P*6_3_′/*m*′*cm*′ and 193.259						
5.6	Transformation to standard setting of MSG	(⅓**a** − ⅓**b**, ⅓**a** + ⅔**b**, **c**; 0, ⅓, 0)						
5.7	Magnetic point group	6′/*m*′*mm*′ (27.5.104)						
5.8	Magnetic unit-cell parameters (Å, °)	**a** = **b** = 17.082, **c** = 4.806						
		α = β = 90, γ = 120						
5.9	MSG symmetry operations (24)	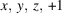			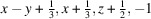			
		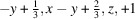			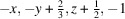			
		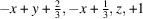			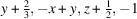			
		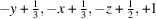			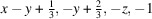			
		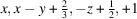			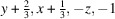			
		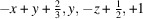			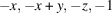			
		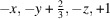			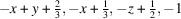			
		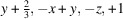			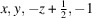			
		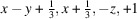			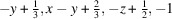			
		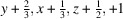			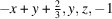			
		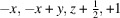			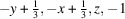			
		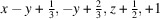			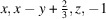			
5.10	MSG symmetry centering operations (3)	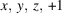						
		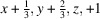						
		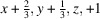						
5.11	Positions (label, *x*, *y*, *z*) of magnetic atoms (2)	Mn1_1	0		0		0	
		Mn1_2	0		0.3333		0	
5.12	Positions (label, *x*, *y*, *z*) of non-magnetic atoms (3)	Ba	0.1111		0.2222		0.75	
		O1	0.0483		0.0966		0.25	
		O2	0.0483		0.4300		0.25	
5.13	Magnetic atom, moment components (m_*x*_, m_*y*_, m_*z*_), symmetry constraints and moment amplitude (|M|, in μ_B_) (1)	Mn1_1	0	0	−3	0, 0, m_*z*_		3
		Mn1_2	0	0	3	0, 0, m_*z*_		3

**Table 6:** Description of the magnetic structure of BaMnO_3_(2H) under its magnetic space group – standard MSG setting See headnote of Table 7 ▸ for the meaning of the symmetry operation outlined in bold. Example of mandatory information in brown.

		BaMnO_3_(2H) magnetic structure (MAGNDATA, No. 1.0.39)						
6.1	Parent space group	*P*6_3_/*mmc* (No. 194)						
6.2	Transformation from parent basis to magnetic structure unit cell	(**a** − **b**, **a** + 2**b**, **c**; 0, 0, 0)						
6.3	Propagation vector	**k** = (⅓, ⅓, 0)						
6.4–6.5	MSG symbol and number	*P*6_3_′/*m*′*cm*′ and 193.259						
6.6	Transformation to standard setting of MSG	(**a**, **b**, **c**; 0, 0, 0)						
6.7	Magnetic point group	6′/*m*′*mm*′ (27.5.104)						
6.8	Magnetic unit-cell parameters (Å, °)	**a** = **b** = 9.8623, **c** = 4.806						
		α = β = 90, γ = 120						
6.9	MSG symmetry operations (24)	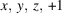			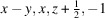			
		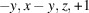			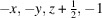			
		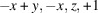			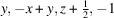			
		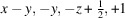			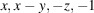			
		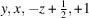			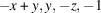			
		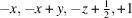			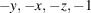			
		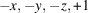			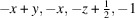			
		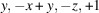			***x*, *y*, −*z* + ½, −1**			
		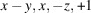			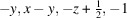			
		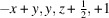			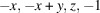			
		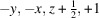			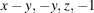			
		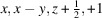			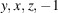			
6.10	MSG symmetry centering operations (1)	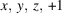						
6.11	Positions (label, *x*, *y*, *z*) of magnetic atoms (2)	Mn1_1	0.3333		0.6667		0	
		Mn1_2	0		0		0	
6.12	Positions (label, *x*, *y*, *z*) of non-magnetic atoms (3)	Ba	0.6667		0		0.25	
		O1	0.3333		0.8116		0.25	
		O2	0.1450		0		0.25	
6.13	Magnetic atom, moment components (m_*x*_, m_*y*_, m_*z*_), symmetry constraints and moment amplitude (|M|, in μ_B_) (1)	Mn1_1	0	0	3	0, 0, m_*z*_		3
		Mn1_2	0	0	−3	0, 0, m_*z*_		3

**Table 7:** Spin basis functions of the two one-dimensional IRs mK_3_ and mK_4_ in the representation analysis of the magnetic ordering of BaMnO_3_(2H) [*P*6_3_/*mmc*, **k** = (⅓, ⅓, 0), Mn on Wyckoff position 2*a*] Mn(1) and Mn(2) are defined here in the parent cell (*P*6_3_/*mmc*) and therefore do not correspond to the splitting shown in Tables 6.11 ▸ and 6.11 ▸. mK_3_ imposes an antiparallel relationship between Mn(1) and Mn(2), which is expressed in the magnetic crystallography description by the MSG symmetry operation 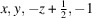 (see Table 6.9 ▸, symmetry operation outlined in bold).

	mK_3_ ψ	mK_4_ ψ
Mn(1) (0, 0, 0) *x*, *y*, *z*	0 0 1	0 0 1
Mn(2) (0, 0, ½) *x*, *y*, −*z* + ½	0 0 −1	0 0 1

**Table 8:** Representation analysis of the magnetic structure of BaMnO_3_ (parent-like setting of the magnetic space group) Primary and secondary modes description (giving the constraints between the magnetic component of the Mn_1 and Mn_2 sites for mK_3_ and mΓ_4_^+^, respectively) obtained with *ISODISTORT*.

		BaMnO_3_
8.1	Description of the primary IR	mK_3_ (two-dimensional) (special direction)
8.2	Description of primary mode(s) and amplitude(s) C_*i*_ (in μ_B_)	mK_3_
		Mn1_1 (0, 0, 1)
		Mn1_2 (0, 0, −0.5)
		C_1_ = 4
8.3	Secondary IR (dimension)	mΓ_4_^+^ (one-dimensional)
8.4	Description of secondary mode(s) and amplitude(s) C_*i*_ (in μ_B_)	mΓ_4_^+^
		Mn1_1 (0, 0, 1)
		Mn1_2 (0, 0, 1)
		C_2_ = −1

**Table 9:** Description of the magnetic structure of TbCrO_3_ (*T*_N3_ < *T* < *T*_N2_) under its MSG Example of mandatory information in brown.

		TbCrO_3_ (*T*_N3_ < *T* < *T*_N2_) (MAGNDATA, No. 0.354)						
9.1	Parent space group	*Pnma*						
9.2	Transformation from parent basis to magnetic unit cell	(**a**, **b**, **c**; 0, 0, 0)						
9.3	Propagation vector	**k** = (0, 0, 0)						
9.4–9.5	MSG symbol and number	*Pn*′*m*′*a* and 62.446						
9.6	Magnetic point group	*m*′*m*′*m* (8.4.27)						
9.7	Magnetic unit-cell parameters (Å, °)	**a** = 5.513, **b** = 7.557, **c** = 5.291						
		α = β = γ = 90						
9.8	Positions (label, *x*, *y*, *z*) of magnetic atoms (2)	Cr	0		0		0.5	
		Tb	0.064		0.25		0.989	
9.9	Positions (label, *x*, *y*, *z*) of non-magnetic atoms (2)	O1	0.470		0.25		0.096	
		O2	0.301		0.049		0.697	
9.10	Magnetic atom, moment components (m_*x*_, m_*y*_, m_*z*_), symmetry constraints and moment amplitude (|M|, in μ_B_) (2)	Cr	0	2.85	0	m_*x*_, m_*y*_, m_*z*_		2.85
		Tb	weak	0	0	m_*x*_, 0, m_*z*_		weak
9.11	Description of the primary IR	mΓ_2_^+^ (one-dimensional)						

**Table 10:** Description of the magnetic structure of TbCrO_3_ under its MSG below *T*_N3_ Example of mandatory information in brown.

		TbCrO_3_ (*T* < *T*_N3_) (MAGNDATA, No. 2.62)						
10.1	Parent space group	*Pnma*						
10.2	Transformation from parent basis to magnetic unit cell	(2**a**, **b**, **c**; 0, 0, 0)						
10.3	Propagation vector	**k**1 = (0, 0, 0)						
		**k**2 = (½, 0, 0)						
10.4–10.5	MSG symbol and number	*Pm*′*n*′2_1_ and 31.127						
10.6	Transformation to standard setting of MSG	(**b**, −**a**, **c**; 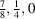 )						
10.7	Magnetic point group	*m*′*m*′2 (7.4.23)						
10.8	Magnetic unit-cell parameters (Å, °)	**a** = 11.026, **b** = 7.557, **c** = 5.291						
		α = β = γ = 90						
10.9	MSG symmetry operations (4)	*x*, *y*, *z*, +1			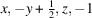			
		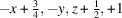			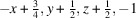			
10.10	MSG symmetry centering operations (1)	*x*, *y*, *z*, +1						
10.11	Positions (label, *x*, *y*, *z*) of magnetic atoms (6)	Cr1_1	0		0		0.5	
		Cr1_2	0.25		0		0	
		Tb1_1	0.03200		0.25000		0.98900	
		Tb1_2	0.21800		0.75000		0.48900	
		Tb1_3	0.96800		0.75000		0.01100	
		Tb1_4	0.28200		0.25000		0.51100	
10.12	Positions (label, *x*, *y*, *z*) of non-magnetic atoms (8)	O1_1	0.23500		0.25000		0.09600	
		O1_2	0.01500		0.75000		0.59600	
		O1_3	0.76500		0.75000		0.90400	
		O1_4	0.48500		0.25000		0.40400	
		O2_1	0.15050		0.04900		0.69700	
		O2_2	0.09950		0.95100		0.19700	
		O2_3	0.84950		0.54900		0.30300	
		O2_4	0.40050		0.45100		0.80300	
10.13	Magnetic atom, moment components (m_*x*_, m_*y*_, m_*z*_), symmetry constraints and moment amplitude (|M|, in μ_B_) (6)	Cr1_1	0	2.85	0	m_*x*_, m_*y*_, m_*z*_		2.85
		Cr1_2	0	−2.85	0	m_*x*_, m_*y*_, m_*z*_		2.85
		Tb1_1	3.17	0	7.76	m_*x*_, 0, m_*z*_		8.38
		Tb1_2	3.17	0	−7.76	m_*x*_, 0, m_*z*_		8.38
		Tb1_3	−3.17	0	−7.76	m_*x*_, 0, m_*z*_		8.38
		Tb1_4	−3.17	0	−7.76	m_*x*_, 0, m_*z*_		8.38

**Table 11:** Representation analysis of the magnetic structure of TbCrO_3_ below *T*_N3_

		TbCrO_3_ (*T* < *T*_N3_)	
11.1	Description of the primary IR	mΓ_2_^+^ (one-dimensional), mX_2_ (two-dimensional)	
11.2	Primary IR mode(s) and amplitudes (C_*i*_ in μ_B_)	mX_2_ mode 1	mΓ_2_^+^ mode 3
		Tb1_1 (1, 0, 0)	Cr1_1 (1, 0, 0)
		Tb1_2 (1, 0, 0)	Cr1_2 (−1, 0, 0)
		Tb1_3 (1, 0, 0)	C_3_ = 0
		Tb1_4 (1, 0, 0)	mΓ_2_^+^ mode 4
		C_1_ = 0	Cr1_1 (0, 1, 0)
		mX_2_ mode 2	Cr1_2 (0, −1, 0)
		Tb1_1 (1, 0, 0)	C_4_ = 2.85
		Tb1_2 (1, 0, 0)	mΓ_2_^+^ mode 5
		Tb1_3 (−1, 0, 0)	Cr1_1 (0, 0, 1)
		Tb1_4 (−1, 0, 0)	Cr1_2 (0, 0, 1)
		C_2_ = 3.17	C_5_ = 0
		mX_2_ mode 3	
		Tb1_1 (0, 0, 1)	
		Tb1_2 (0, 0, −1)	
		Tb1_3 (0, 0, 1)	
		Tb1_4 (0, 0, −1)	
		C_3_ = 0	
		mX_2_ mode 4	
		Tb1_1 (0, 0, 1)	
		Tb1_2 (0, 0, −1)	
		Tb1_3 (0, 0, −1)	
		Tb1_4 (0, 0, 1)	
		C_4_ = 7.76	
11.3	Description of the secondary IR	mΓ_1_^−^ (one-dimensional) (not present in the model)	
